# Parenchyma-Sparing Strategy in Central Non-Small Cell Lung Cancer: An Institutional Experience on Surgical Selection, Complications, and Survival

**DOI:** 10.3390/cancers18203340

**Published:** 2026-10-16

**Authors:** Cornel Florentin Savu, Andrei Cristian Bobocea, Amir-Andrei Sabha, Camelia-Alexandra Paruschi, Alina-Elena Tucana, Antoaneta Pungă, Silviu Gabriel Vlăsceanu

**Affiliations:** 1 Thoracic Surgery I, University of Medicine and Pharmacy “Carol Davila”, 020021 Bucharest, Romania; drsavu25@yahoo.com; 2 Department of Thoracic Surgery, “Marius Nasta” National Institute of Pneumology, 050150 Bucharest, Romania; andrei.bobocea@gmail.com (A.C.B.); amirandreisabha25@gmail.com (A.-A.S.); alina-elena.tucana0226@rez.umfcd.ro (A.-E.T.); silviu.vlasceanu@drd.umfcd.ro (S.G.V.); 3 Medical Physiology Discipline, Functional Sciences Department, Faculty of Medicine, University of Medicine and Pharmacy “Carol Davila”, 020021 Bucharest, Romania

**Keywords:** non-small cell lung cancer, central lung cancer, sleeve lobectomy, pneumonectomy, parenchyma-sparing surgery, anastomotic safety, nodal status, overall survival

## Abstract

Background/Objectives: Sleeve lobectomy serves as a parenchyma-sparing alternative to pneumonectomy for central non-small cell lung cancer (NSCLC). This study describes and compares early postoperative outcomes and overall survival between sleeve lobectomy and pneumonectomy and explores factors associated with survival. Methods: A retrospective cohort analysis was conducted on 97 consecutive patients with central NSCLC operated at the Marius Nasta National Institute of Pneumophthisiology between September 2021 and December 2022, with follow-up through 1 March 2025. Patients were divided into two groups: sleeve lobectomy (*n* = 41) and pneumonectomy (*n* = 56). Early morbidity, mortality, and overall survival were evaluated using univariate analysis and multivariable Cox proportional hazards regression. Results: After multivariable adjustment, surgical procedure was not significantly associated with overall survival. A low rate of major anastomotic complications was observed in the sleeve lobectomy group, with one case of early partial anastomotic leak (2.44%) requiring surgical reinforcement. In an exploratory multivariable model, pathological nodal involvement and the number of involved lymph nodes were associated with mortality. Conclusions: In appropriately selected patients where an R0 parenchyma-sparing resection is technically feasible, sleeve lobectomy is associated with acceptable morbidity and survival. After adjustment, the surgical procedure was not significantly associated with overall survival. Nodal disease burden was the factor most strongly associated with survival in this cohort.

## Simple Summary

Surgery for centrally located lung tumors often requires the complete removal of a lung, a procedure that severely reduces a patient’s breathing capacity and overall quality of life. A tissue-saving alternative exists, where only the affected lung lobe and a section of the main airway are removed and carefully reconstructed. However, the long-term success of this lung-sparing approach compared to complete lung removal remains debated in daily clinical practice. We analyzed patients treated at our institution to describe and compare early surgical outcomes and overall survival after these two procedures. In selected patients, lung-sparing surgery was associated with acceptable recovery and survival. Survival was most strongly associated with lymph node involvement rather than the type of resection, although the two groups were not directly comparable. These observational findings require confirmation in larger studies.

## 1. Introduction

Lung cancer remains the leading cause of cancer-related mortality worldwide, with non-small cell lung cancer accounting for approximately 85% of all diagnosed cases. Centrally located tumors present a unique surgical challenge due to their immediate proximity to major vascular structures, the pulmonary artery, and the main tracheobronchial tree. Historically, total pneumonectomy was considered the definitive interventional standard required to achieve complete anatomical radicality and negative surgical margins for central malignancies. However, the complete sacrifice of a functioning lung induces immediate loss of respiratory reserve, increased pulmonary vascular resistance, and severe long-term functional impairment. Furthermore, preserving functional lung parenchyma has been consistently linked to superior post-operative quality of life and exercise tolerance [1].

To mitigate these severe functional sequelae, parenchyma-sparing techniques were introduced into thoracic practice. Early foundational work by Thomas in 1956 [2] and Paulson and Shaw in 1955 [3] demonstrated the technical feasibility of bronchial anastomoses and bronchoplastic procedures to preserve functional lung tissue. Over subsequent decades, sleeve lobectomy evolved from a procedure reserved for localized or low-grade endobronchial lesions into a primary therapeutic modality for aggressive central carcinomas.

Contemporary evidence from large multicenter series and nationwide audits has further supported the clinical utility of parenchyma-sparing surgery. Comparative studies have reported that sleeve lobectomy can replace pneumonectomy in selected patients, with comparable or better survival and lower early morbidity and perioperative mortality [4,5]. Despite growing clinical adoption, debates persist regarding the oncological outcomes of sleeve lobectomy compared to pneumonectomy in real-world cohorts characterized by advanced stage or regional nodal involvement. Furthermore, surgical decision-making is often confounded by baseline stage imbalance between surgical arms. The objective of this retrospective study is to describe and compare the early clinical outcomes, complication profiles, and long-term overall survival of patients undergoing sleeve lobectomy versus total pneumonectomy for central non-small cell lung cancer at a high-volume tertiary thoracic surgery center, and to outline our institutional approach to surgical selection, recognizing that the two groups are not directly comparable.

## 2. Materials and Methods

### 2.1. Study Design and Patient Population

This retrospective, observational, longitudinal, and non-randomized cohort study evaluated a sample of 97 patients diagnosed with lung cancer who underwent surgical resection in a high-volume tertiary referral center. All clinical, pathological, and surgical data were secondary data retrieved from the Thoracic Surgery Department at the “Marius Nasta” Institute of Pneumophthisiology in Bucharest, Romania. The study inclusion period spanned from September, 2021 to December, 2022, with the final cross-sectional data collection and follow-up cutoff established on 1 March 2025. The study protocol was conducted in accordance with the Declaration of Helsinki and was formally approved by the institutional ethics committee of the “Marius Nasta” Institute (No. 22062/27.09.2021).

### 2.2. Preoperative Diagnostic Workup, Staging Modalities, and Multimodal Correlation

All patients included in the study underwent a standardized, comprehensive preoperative diagnostic and staging evaluation to assess both anatomical and oncological resectability as well as cardiopulmonary reserve, in accordance with current ESMO Clinical Practice Guidelines for early and locally advanced NSCLC [6]. The diagnostic pathway integrated contrast-enhanced chest and abdominal computed tomography (CT), dedicated contrast-enhanced brain magnetic resonance imaging (MRI), positron emission tomography–computed tomography (^18^F-FDG PET-CT), baseline pulmonary function testing (PFTs), and flexible bronchoscopy.

#### 2.2.1. Computed Tomography and Morphological Assessment

Contrast-enhanced thoracic and upper abdominal CT scans (1.25 mm slice thickness) served as the initial morphological imaging modality. CT evaluated primary tumor diameter, central endobronchial growth, relationship to the tracheobronchial tree, and macrovascular invasion of the pulmonary artery or pulmonary veins (Figure 1). Mediastinal and hilar lymph nodes with a short-axis diameter exceeding 10 mm were classified as morphologically suspicious for metastatic involvement (cN+).

#### 2.2.2. Brain Magnetic Resonance Imaging and Central Nervous System Staging

Contrast-enhanced brain magnetic resonance imaging (MRI) was systematically performed in all candidates as the primary modality to exclude occult central nervous system metastases, in accordance with ESMO guidelines for resectable NSCLC. In cases where MRI was clinically contraindicated (e.g., non-compatible metallic implants or severe claustrophobia), contrast-enhanced head computed tomography was utilized as an alternative staging method.

#### 2.2.3. Positron Emission Tomography and Nodal Staging

Whole-body ^18^F-FDG PET-CT was utilized for metabolic staging, assessment of mediastinal lymph node status, and exclusion of occult distant metastases according to ESMO recommendations [6]. PET-CT demonstrated high sensitivity for detecting regional nodal involvement, leading to stage re-classification (upstaging or downstaging) in patients compared to CT alone. Non-FDG-avid CT-enlarged lymph nodes were confirmed negative, allowing curative-intent resections, whereas FDG-avid N2/N3 nodes prompted invasive mediastinal staging (endobronchial ultrasound-guided transbronchial needle aspiration [EBUS-TBNA] or cervical mediastinoscopy) to exclude unresectable disease prior to surgery.

#### 2.2.4. Functional Evaluation and Cardiopulmonary Reserve

Pulmonary function testing, including spirometry and diffusion capacity for carbon monoxide (DLCO), was conducted in all candidates following institutional functional stratification protocols for endobronchial tumor resections [7]. Predicted postoperative forced expiratory volume in 1 s (ppoFEV_1_%) and predicted postoperative DLCO (ppoDLCO%) were routinely calculated based on the number of functioning lung segments to be resected. Patients with marginal functional reserve (ppoFEV_1_ or ppoDLCO between 30% and 40%) underwent cardiopulmonary exercise testing (CPET) to measure peak oxygen consumption (VO_2_max). A low predicted functional reserve (ppoFEV_1_ < 40% or VO_2_max < 15 mL/kg/min) represented a major clinical driver prioritizing sleeve lobectomy over pneumonectomy to avoid post-resection respiratory failure or severe pulmonary hypertension.

#### 2.2.5. Bronchoscopic Evaluation and Airway Mapping

Preoperative flexible bronchoscopy was systematically performed in all patients to visually map the endobronchial extent of the tumor using both standard white-light bronchoscopy (WLB) and autofluorescence/fluorescence imaging (Olympus Corporation, Tokyo, Japan) (Figure 2). Fluorescence imaging was used to enhance the contrast of neoplastic tissue and submucosal tumor margins, helping to determine the precise distance from the primary lesion to the main carina and lobar orifices. In cases with central lesions originating from lobar orifices, such as left upper lobe tumors extending into the distal half of the left main bronchus, bronchoscopy delineated the wide-base implantation along the bronchial wall while verifying that the distal airway to the lower lobe remained uninvolved. Identifying a proximal tumor-free mucosal margin of at least 5 to 10 mm during endoscopy aided in the initial planning of a parenchyma-sparing sleeve lobectomy, although final microscopic submucosal clearance was strictly established via intraoperative frozen section analysis [8].

#### 2.2.6. Diagnostic Correlation and Multidisciplinary Decision-Making

The final surgical strategy was established through a multidisciplinary thoracic oncology board review, correlating anatomical mapping (CT and bronchoscopy), metabolic nodal status (PET-CT), and functional tolerances (PFTs). Discrepancies between CT and bronchoscopy regarding proximal bronchial clearance were resolved in favor of direct bronchoscopic visualization. Similarly, metabolic nodal staging via PET-CT served as the gatekeeper for functional eligibility: patients with N0-N1 disease on PET-CT and borderline lung function were directed toward sleeve lobectomy, whereas patients requiring pneumonectomy based on anatomical extent were only cleared for surgery if both metabolic M0 status and robust cardiopulmonary parameters were met.

### 2.3. Surgical Techniques and Cohort Stratification

Surgical interventions were performed via standard open thoracotomy, prioritizing parenchyma-sparing techniques whenever anatomically and oncologically feasible to achieve microscopically negative margins (R0 resection). The study sample was stratified into two distinct surgical cohorts:

- Sleeve Lobectomy (SL) Group: This cohort comprised patients who underwent anatomical lobectomy combined with a circumferential, segmental resection of the bronchial axis (“bronchial sleeve”) followed by a primary end-to-end tracheobronchial airway reconstruction. Within this group, cases involving tumor infiltration into the pulmonary vascular tree were managed via concurrent segmental resection of the main pulmonary artery axis followed by a vascular end-to-end anastomosis, completing a pulmonary arterioplasty (combined bronchovascular sleeve).
- Pneumonectomy (PN) Group: This cohort included patients in whom parenchyma preservation was technically or oncologically impossible due to extensive structural invasion of the pulmonary hilum, multi-lobar involvement, or the inability to secure disease-free margins via a sleeve procedure, thereby requiring a total pneumonectomy.

### 2.4. Inclusion and Exclusion Criteria

To ensure a highly homogenous study population and minimize confounding variables associated with multimodal treatment protocols or non-standard surgical reconstructions, strict selection criteria were applied to the entire patient pool.

#### 2.4.1. Inclusion Criteria

Patients were required to meet all of the following criteria to be eligible for analysis:

- Age ≥ 18 years.
- Histologically confirmed non-small cell lung cancer (NSCLC) classified within upfront surgical curative-intent stages (specifically AJCC/IASLC Stages IA through IIIA resectable disease).
- Surgical management consisting of either an anatomical sleeve lobectomy (with or without associated pulmonary arterioplasty) or a total pneumonectomy performed as the primary, definitive treatment.

#### 2.4.2. Exclusion Criteria

Patients presenting with any of the following criteria were excluded from the study:

- Receipt of any modality of neoadjuvant (induction) therapy, including preoperative chemotherapy, radiation therapy, or combined chemoradiotherapy.
- Isolated bronchial sleeve resections performed with complete preservation of the lung parenchyma (i.e., bronchoplastic segmental resections without associated anatomical lobectomy).
- Complex tracheobronchial resections involving the main carina (carinal pneumonectomies or major carinal reconstructions).
- Low-grade neuroendocrine tumors (typical and atypical carcinoids) or benign endobronchial lesions, as the present study focused strictly on primary invasive NSCLC.

### 2.5. Surgical Techniques and Institutional Experience

Sleeve lobectomy was performed via open thoracotomy under single-lung ventilation. Vascular control was routinely secured prior to bronchial transection, and intraoperative frozen section analysis was systematically utilized to guarantee microscopically clear margins. When pulmonary artery involvement was identified, a combined bronchovascular sleeve resection was performed; vascular reconstruction was accomplished either through tangential arterial resection with primary or patch repair, or via complete segmental arterial resection followed by an end-to-end vascular anastomosis.

In the surgical literature, four main bronchial anastomotic techniques are described for airway reconstruction: simple interrupted sutures across the entire circumference, a continuous running suture for the posterior membranous wall combined with simple interrupted sutures for the cartilaginous ring, a single continuous running suture for the entire anastomosis, and two continuous running sutures for the membranous and cartilaginous portions separately. In our institutional practice, airway reconstruction during sleeve lobectomy was standardized using the hybrid technique: a continuous running suture for the membranous posterior wall and simple interrupted sutures for the cartilaginous framework, using 4-0 polydioxanone monofilament (SMI AG, St. Vith, Belgium) with all knots tied extraluminally. To mitigate the risk of anastomotic dehiscence and bronchopleural fistula, the reconstructed suture line was systematically reinforced with an autologous vascularized tissue flap, preferentially a pericardial fat pad or an intercostal muscle flap. Systematic mediastinal lymph node dissection was carried out in all cases.

Total pneumonectomy was reserved strictly for cases where sleeve lobectomy was technically or oncologically unfeasible due to extensive hilar structural invasion or multi-lobar disease. Interventions were executed via a standard fifth-intercostal-space thoracotomy. Individual hilar control was achieved through sequential vascular ligation or stapling, followed by proximal mainstem bronchial transection close to the carina. Mainstem bronchial stump closure was executed either mechanically using an endo-GIA stapler (Medtronic, Minneapolis, MN, USA) or manually. Manual closure for both right and left mainstem bronchial stumps involved a continuous running suture followed by a second layer of locked continuous running suture to ensure airtight closure. Unlike sleeve lobectomies, chest tube placement was omitted postoperatively in standard pneumonectomies to maintain intrapleural pressure dynamics and prevent acute mediastinal shift.

### 2.6. Statistical Analysis

Statistical analysis was performed using R statistical software, version 4.2.3 (R Core Team, 2023; R Foundation for Statistical Computing, Vienna, Austria; available at https://www.R-project.org, accessed on 1 March 2025). Comprehensive data summarization, survival modeling, and high-quality clinical visualizations were executed utilizing the specialized ecosystem packages: gtsummary [9], survival [10] and survminer [11].

The primary endpoint of the study was overall survival (OS), defined as the time interval from the date of the primary surgical intervention to documented death; patients alive on 1 March 2025 were censored at that date. Vital status was ascertained from institutional electronic health records, national civil registry databases, and direct telephone follow-up. Survival was estimated with the Kaplan–Meier method and summarized as median survival with 95% confidence intervals and as 1-, 2-, and 3-year survival probabilities. Median follow-up was estimated with the reverse Kaplan–Meier method. Survival curves were compared with the log-rank test. Thirty-day and 90-day mortality were calculated as crude proportions. To explore factors associated with overall survival, a multivariable Cox proportional hazards model was fitted including age, sex, surgical procedure, histological subtype, pathological T stage, pathological N stage, tumor diameter, and number of involved lymph nodes, entered simultaneously; no automated variable selection was used. Analyses were performed on complete cases (8 patients with missing covariates were excluded). Categorical variables were compared with Pearson’s chi-squared or Fisher’s exact test, continuous variables with Welch’s t-test, and postoperative outcomes with Fisher’s exact test. A two-sided *p*-value < 0.05 was considered statistically significant. With 67 events for 12 estimated parameters, the model was considered exploratory.

## 3. Results

### 3.1. Baseline Demographic and Clinical Characteristics

The final study population comprised 97 consecutive patients diagnosed with central non-small cell lung cancer in a surgically resectable stage, who underwent operative intervention within the Thoracic Surgery Department at the Marius Nasta Institute of Pneumophthisiology. Of these, 41 patients (42.3%) underwent parenchyma-sparing surgery and were assigned to the Sleeve Lobectomy (SL) group, while 56 patients (57.7%) required radical total resection and formed the Pneumonectomy (PN) group.

Baseline demographic parameters were highly comparable between the two cohorts. The mean age of the entire cohort was 58.00 ± 9.93 years, with no statistically significant difference observed between the SL group (58.95 ± 12.19 years) and the PN group (57.30 ± 7.92 years, *p* = 0.45). Sex distribution was identical across both subsamples, demonstrating a pronounced male predominance (73% in both groups, *p* = 0.99). Similarly, histopathological subtype distribution was statistically uniform (*p* = 0.78); squamous cell carcinoma was the most prevalent variant overall (46%), accounting for 49% of cases in the SL group and 45% in the PN group, followed by adenocarcinoma (38% overall; 34% in SL vs. 41% in PN).

In contrast, central tumor topography and structural invasion profiles led to significant differences in local tumor extension between the cohorts. The clinical distribution of primary T status varied significantly (*p* = 0.017), with the PN cohort demonstrating a higher concentration of locally advanced configurations (T3: 38%; T4: 13%). Conversely, the SL group was predominantly characterized by early-stage local extension (T1: 32%; T2: 44%). Although the mean tumor diameter was larger in the PN group (5.25 ± 2.46 cm) than in the SL group (4.67 ± 1.76 cm), this specific morphologic metric did not reach formal statistical significance (*p* = 0.20).

Pathological nodal staging (N status) was statistically comparable between the two surgical approaches (*p* = 0.55); however, sleeve lobectomy was more frequently performed in node-negative cases (N0: 39% vs. 30%), whereas pneumonectomy exhibited a higher raw proportion of N2 multi-level mediastinal involvement (45% vs. 34%). Regarding lymphadenectomy metrics, the mean number of harvested lymph nodes was significantly higher in the PN group (5.67 ± 2.67) than in the SL group (4.67 ± 1.90, *p* = 0.035). However, the mean number of pathologically invaded (metastatic) lymph nodes did not differ significantly between the two cohorts (1.76 ± 2.54 for PN vs. 1.17 ± 1.51 for SL, *p* = 0.168). All baseline demographic and clinico-pathological comparisons are summarized in Table 1.

### 3.2. Surgical Specifics and Anastomotic Techniques

Within the sleeve lobectomy cohort (*n* = 41), all patients underwent anatomical parenchymal resection combined with airway reconstruction. Right-sided sleeve lobectomies were performed in 28 cases (predominantly right upper sleeve lobectomies, *n* = 22), while left-sided sleeve lobectomies accounted for 13 cases (all left upper sleeve lobectomies).

Reconstructive approaches were tailored to the precise anatomical boundaries of the structural invasion as follows:

- Simple bronchial sleeve anastomosis (end-to-end reconstruction of the bronchial tree without vascular involvement): 28 cases.
- Combined bronchovascular sleeve (segmental bronchial resection combined with complete segmental arterial resection and end-to-end vascular anastomosis): 8 cases.
- Tangential pulmonary arterioplasty/Patch angioplasty: 5 cases.

Overall, pulmonary artery reconstruction was performed in 13 patients (8 left-sided and 5 right-sided procedures).

In the pneumonectomy cohort (*n* = 56), 32 procedures were performed on the right side and 24 on the left side. Mainstem bronchial stump closure was accomplished using a mechanical endo-stapler in 40 cases and manual double-layer continuous suturing in 16 cases.

### 3.3. Postoperative Complications and Timeline Analysis

#### 3.3.1. Immediate Postoperative Period (Days 0 to 7)

Immediate morbidity was dominated by respiratory and cardiac events. In the sleeve lobectomy group, acute retention of secretions requiring therapeutic flexible bronchoscopy occurred in 2 patients (4.88%). No immediate disruption or mechanical failure of the airway anastomosis was observed during these procedures. New-onset atrial fibrillation within the first week occurred in 4 sleeve patients (9.76%) and 14 pneumonectomy patients (25.00%).

#### 3.3.2. Early Postoperative Period (Days 8 to 30)

One patient in the sleeve lobectomy group (2.44%) presented with an early partial anastomotic leak on postoperative day 12, which was managed via re-thoracotomy and muscle flap reinforcement (Clavien–Dindo Grade IIIb). In the pneumonectomy group, early severe complications included mainstem bronchial stump fistula in 3 patients (5.36%), acute post-pneumonectomy pulmonary edema in 3 patients (5.36%), and contralateral pulmonary atelectasis in 2 patients (3.57%).

#### 3.3.3. Late Postoperative Period (Beyond 3 Months)

In the sleeve lobectomy cohort, late airway complications were restricted to localized anastomotic bronchial stenosis in 2 patients (4.88%), both occurring at the hybrid suture line between 4 and 6 months postoperatively; both were successfully managed via endoscopic balloon dilation without requiring re-intervention. Severe functional restriction was noted in 1 patient (2.44%). In the pneumonectomy cohort, late morbidity included post-pneumonectomy syndrome with severe respiratory failure in 4 patients (7.14%) and chronic post-pneumonectomy empyema in 2 patients (3.57%). A detailed distribution of all postoperative adverse events categorized according to the Clavien–Dindo classification system is presented in Table 2.

### 3.4. Survival and Mortality Analysis

The survival analysis of the entire study population highlighted the severe oncological impact of central bronchopulmonary carcinoma, with numerically different survival between the two surgical groups (Table 3).

The primary endpoint of overall survival was quantified utilizing median survival and Restricted Mean Survival Time (RMST). For the global cohort, the RMST was 53.80 months. When stratified by surgical procedure, the RMST was 63.80 months for the sleeve lobectomy group and 46.40 months for the pneumonectomy group. Median survival was 39.00 months (95% CI: 33.00 to N/A) for the sleeve lobectomy group and 28.50 months (95% CI: 20.00 to 45.00) for the pneumonectomy group. The log-rank comparison indicated that this difference was at the limit of statistical significance (*p* = 0.061).

Early 30-day postoperative mortality occurred in 4 patients (4.12% of the total cohort), comprising 1 patient (2.44%) in the sleeve lobectomy group and 3 patients (5.36%) in the pneumonectomy group. Primary causes of early postoperative mortality were restricted to acute cardio-respiratory events, specifically acute respiratory distress syndrome/severe pulmonary edema (*n* = 1 in sleeve lobectomy, *n* = 2 in pneumonectomy) and acute heart failure (*n* = 1 in pneumonectomy).

Across the complete follow-up period, late mortality was predominantly driven by oncological disease progression rather than surgical complications. Systemic relapse and distant metastatic progression accounted for 82.6% of all recorded late deaths in both patient groups.

Overall mortality increased with pathological stage: 36.8% (7 of 19) for Stage I, 65.0% (26 of 40) for Stage II, and 94.7% (36 of 38) for Stage III disease. When evaluated by primary histological subtype, overall mortality was 78.4% (29 of 37) for adenocarcinoma, 62.2% (28 of 45) for squamous cell carcinoma, and 80.0% (12 of 15) for other histologic variants, showing no statistically significant difference across cell types (*p* = 0.270).

### 3.5. Margin Status (R0 vs. R1) and 3-Year Recurrence Patterns

Complete radical resection (R0) was achieved in the majority of patients across both surgical cohorts. In cases with microscopic residual tumor (R1), failure patterns were closely linked to the specific anatomical site of involvement: microscopic bronchial involvement (R1-B+) primarily trended towards local anastomotic recurrence, whereas microscopic vascular involvement (R1-V+) was associated with local-regional failure. Combined broncho-vascular residual disease (R1-B+ and R1-V+) demonstrated the highest observed proportion of local failure and overall mortality, although formal hazard ratios were omitted due to the small sample size of R1 cases. At 3-year post-operative follow-up, the overall recurrence rate for the study population was 48.4% (47 of 97). Progression was predominantly driven by distant systemic recurrence (37.1%), whereas isolated local or anastomotic recurrences occurred in only 11.3% of cases. There was no statistically significant difference in 3-year overall recurrence rates between the sleeve lobectomy group (43.9%) and the pneumonectomy group (51.8%, *p* = 0.44); the study was not designed or powered to assess local disease control.

### 3.6. Multivariable Cox Regression Analysis for Overall Survival

To explore factors associated with overall survival, a multivariable Cox proportional hazards regression model was fitted, incorporating age, sex, surgical procedure, histological subtype, pathological T stage, pathological N stage, tumor diameter, and the number of involved lymph nodes simultaneously. Out of the initial study cohort of 97 patients, 8 patients were excluded from the regression analysis due to incomplete clinico-pathological data or missing baseline covariates, yielding a final analytical sample of 89 evaluable patients with complete survival tracking (37 in the Sleeve Lobectomy group and 52 in the Pneumonectomy group), among whom 67 death events were recorded (Table 4).

Demographic factors including age (HR = 1.00, 95% CI: 0.97–1.03, *p* = 0.831) and male sex (HR = 1.15, 95% CI: 0.56–2.39, *p* = 0.702) were not significantly associated with overall survival. Histological subtype (squamous cell carcinoma vs. adenocarcinoma: HR = 0.78, *p* = 0.462) and primary pathological T-stage (*p* > 0.05 across all T-subcategories) were likewise not significantly associated with mortality.

The type of surgical resection (pneumonectomy vs. sleeve lobectomy) was not significantly associated with death (HR = 1.31, 95% CI: 0.74–2.31, *p* = 0.349). This suggests that the observed survival difference may reflect baseline anatomical and oncological differences rather than the surgical procedure itself, although residual confounding cannot be excluded. In contrast, pathological nodal status and the number of involved lymph nodes were the variables most strongly associated with overall survival in this model. Pathological N1 involvement was associated with a more than two-fold higher hazard of death compared with N0 disease (HR = 2.46, 95% CI: 1.12–5.40, *p* = 0.024). Pathological N2 involvement was associated with an approximately doubled hazard (HR = 2.05, 95% CI: 1.01–4.30, *p* = 0.050), at the boundary of statistical significance. Each additional involved lymph node was associated with a 15% higher hazard of death (HR = 1.15 per positive node, 95% CI: 1.03–1.30, *p* = 0.017).

Importantly, the simultaneous inclusion of pathological N stage and the absolute number of involved lymph nodes in this model introduces a high risk of collinearity, as these variables describe closely related aspects of nodal disease [12]. Similarly, T-stage and tumor diameter may partially overlap. Consequently, the respective hazard ratios for these variables should be interpreted strictly as exploratory associations reflecting the overall nodal and local disease burden, rather than definitively independent prognostic determinants.

### 3.7. Proposed Institutional Conceptual Framework for Surgical Selection in Central NSCLC

To contextualize our clinical observations within daily surgical practice, we present a structured, three-pillar conceptual framework reflecting our institutional approach to guide the choice between sleeve lobectomy and pneumonectomy in central NSCLC (Figure 3). This framework reflects our institutional practice of prioritizing lung parenchymal preservation when an R0 resection is considered feasible; it was not derived from, or validated by, the present data.

#### 3.7.1. Functional Criteria (Cardiopulmonary Reserve)

Sleeve Lobectomy Prioritization: In our practice, this approach is favored in patients with marginal functional reserve (low ppoFEV_1_ or ppoDLCO) to reduce the risk of postoperative respiratory failure or right heart failure. In functionally fit patients, it is also preferred when technically and oncologically feasible, based on the physiological rationale that lung preservation may better maintain functional capacity and quality of life; these outcomes were not measured in the present study.

Pneumonectomy Reserve: Considered in patients with adequate cardiopulmonary reserve (ppoFEV_1_ > 40%, VO_2_max > 15 mL/kg/min) in whom sleeve reconstruction is technically or oncologically not feasible.

#### 3.7.2. Anatomical and Lesional Criteria

- Airway and Vascular Mapping: In our practice, sleeve lobectomy is considered whenever a microscopically negative margin (R0) is expected at both the bronchial transection line and the pulmonary artery. Combined bronchovascular reconstructions or tangential arterial patch repairs are preferred over pneumonectomy when vascular involvement is localized.
- Primary Indications for Pneumonectomy: In our practice, performed when the tumor crossed the interlobar fissure involving adjacent lobes, in cases of massive, fixed hilar invasion extending to the origin of the main pulmonary artery, or when an R0 margin could not be expected with sleeve repair.

#### 3.7.3. Histopathological and Nodal Burden Criteria

While low-grade endobronchial lesions (e.g., typical/atypical carcinoids) represent classic indications for pure bronchial sleeve resection, in our cohort, patients with squamous cell carcinoma or adenocarcinoma treated with sleeve lobectomy had survival that was not significantly different from pneumonectomy after adjustment (Section 3.6); this study cannot establish oncological equivalence.

From a surgical decision-making standpoint, clinical node-negative (cN0) or hilar nodal (cN1) status on preoperative staging favored upfront parenchyma-sparing sleeve resection. While clinically overt mediastinal nodal involvement (cN2) typically warrants multidisciplinary consideration for induction systemic therapy, upfront sleeve lobectomy was only considered in highly selected patients with discrete, non-bulky nodal disease where complete anatomical R0 clearance and systematic mediastinal lymphadenectomy were technically achievable. Postoperatively, pathological nodal status (pN) serves as the primary prognostic tool guiding adjuvant therapy. In our exploratory multivariable analysis, pathological nodal burden (HR = 1.15 per involved node, *p* = 0.017), rather than the extent of parenchymal resection (sleeve lobectomy vs. pneumonectomy, *p* = 0.349), was the key factor associated with overall survival.

## 4. Discussion

The primary imperative in the surgical management of centrally located NSCLC is achieving complete anatomical radicality (R0 resection) while optimizing postoperative quality of life and functional reserve. While sleeve lobectomy (SL) was historically reserved for low-grade endobronchial lesions or typical/atypical carcinoids—where lung parenchyma preservation is paramount [13]—our study illustrates its use in primary epithelial carcinomas. In our series of 97 patients—84% of whom presented with squamous cell carcinoma or adenocarcinoma—sleeve lobectomy was feasible, demonstrating acceptable early outcomes and survival, although the study cannot establish oncological control relative to pneumonectomy.

### 4.1. Parenchymal Sparing, Technical Feasibility, and Mortality

Preserving functioning lung tissue is expected to mitigate the physiological consequences of pneumonectomy (PN). Although bronchial sleeve reconstruction is technically more demanding—requiring meticulous anastomotic techniques (single continuous, double continuous, or interrupted sutures) and occasional combined pulmonary vascular reconstruction—previous reviews of airway reconstruction describe several anastomotic techniques (interrupted, continuous, or combined sutures) with low rates of anastomotic breakdown, without clear evidence that one technique is superior [14].

In unadjusted analysis, median survival was numerically longer in the sleeve group (39.0 vs. 28.5 months; log-rank *p* = 0.061) and crude mortality was numerically lower (60.97% vs. 78.57%); these differences were not statistically significant and are likely influenced by baseline imbalance in tumor extent (Section 4.2). Previous studies [15,16,17] reported that lung-sparing resections did not compromise survival compared with pneumonectomy when R0 status was achieved, although our follow-up does not allow comparison of 5-year outcomes.

### 4.2. Deconstruction of Selection Bias: Stage vs. Surgical Technique

While univariate Kaplan–Meier estimates indicated a trend toward superior overall survival in the sleeve cohort (*p* = 0.061), Cox proportional hazards modeling showed that the surgical procedure itself was not significantly associated with death (HR = 1.31, 95% CI: 0.74–2.31, *p* = 0.349). Although squamous cell carcinoma was the most prevalent histotype in our cohort and was not independently associated with mortality in the multivariable model, detailed histological subtyping and architectural patterns in invasive squamous cell lung cancer have been documented to play an important role in shaping patient survival [18].

This pattern is compatible with the selection bias inherent to real-world surgical decision-making. Consistent with prior reports [19] and our own findings, the choice to perform a pneumonectomy was predominantly dictated by unfavorable local anatomical constraints:

1. Primary Tumor Burden: Patients undergoing PN more often had locally advanced primary tumors (T3/T4 in 50.0% of PN cases vs. 24.4% in SL; *p* = 0.017 for the overall T-category distribution), although mean tumor diameter did not differ significantly (*p* = 0.20).
2. Nodal Burden: A numerically higher proportion of PN patients had pathological N2 disease (45% vs. 34%), although the overall N-category distribution did not differ significantly (*p* = 0.55).

Thus, the observed survival difference between SL and PN may largely reflect baseline disease burden rather than the procedure itself, although residual confounding cannot be excluded. Because pneumonectomy was strictly reserved for anatomically extensive or multilobar disease, the two surgical groups cannot be considered directly comparable treatment alternatives due to strong confounding by indication. The absence of a statistically significant difference in our multivariable Cox model does not establish equivalent survival, particularly given the wide confidence interval (HR 1.31, 95% CI 0.74–2.31), which is compatible with both no difference and a clinically relevant excess hazard with pneumonectomy.

In our study, patients receiving neoadjuvant therapy were deliberately excluded to maintain cohort homogeneity regarding primary surgical outcomes. Hypothetically, if patients undergoing induction chemotherapy had been included, downstaging of centrally invasive tumors might have enabled conversion from an initial pneumonectomy plan to a parenchyma-sparing sleeve lobectomy. However, neoadjuvant treatment may induce tissue fibrosis and vascular fragility, which could increase technical difficulty and the risk of anastomotic complications. By focusing exclusively on upfront surgical candidates, our findings describe outcomes of primary resections without the potential confounding effect of preoperative systemic therapy: sleeve lobectomy was associated with acceptable survival and a low rate of major anastomotic complications (one partial leak, Clavien–Dindo grade IIIb).

### 4.3. Postoperative Morbidity and Complication Profiles

The spectrum of postoperative complications differed between the two cohorts, echoing findings from prior series [20,21]. Numerically, pneumonectomy patients had more bronchial stump fistulas (5.4% vs. 0%), chronic empyema (3.6% vs. 0%), and new-onset atrial fibrillation (25.0% vs. 9.8%). Overall morbidity (39.0% vs. 48.2%; *p* = 0.41) and 30-day mortality (2.4% vs. 5.4%; *p* = 0.64) did not differ significantly (Fisher’s exact test), and the study was not powered to compare individual complications.

In major thoracic surgical literature, post-pneumonectomy BPF rates typically range between 4% and 12%, which aligns closely with our observed rate of 5.4%. When postpneumonectomy bronchial stump disruption occurs, prompt surgical re-exploration via early ipsilateral thoracotomy and stump reinforcement represents a key therapeutic salvage strategy to prevent catastrophic pleural sepsis [22]. Conversely, international benchmarks for sleeve resections report anastomotic complication rates ranging from 2% to 6% [20,21]. In our sleeve lobectomy cohort, no bronchopleural fistulas or complete structural anastomotic breakdowns occurred (0.0%). Anastomotic complications comprised one early partial leak (2.44%), managed surgically, and two late stenoses (4.88%) treated by endoscopic balloon dilation, i.e., 3 of 41 patients (7.3%) overall. This low rate may reflect patient selection and standardized technique; the study design does not allow these contributions to be separated.

Sleeve lobectomy complications in our series were predominantly localized to transient airway management issues, including sputum retention and segmental atelectasis, managed with chest physiotherapy (grade I, *n* = 4) or therapeutic bedside bronchoscopy (grade IIIa, *n* = 2). Our institutional protocol includes flexible bronchoscopy at 3 to 4 weeks postoperatively to screen for anastomotic ischemia, granulation tissue, or early luminal stenosis.

### 4.4. Technical Nuances and Anastomotic Safety Rationale

In this series, the standardized hybrid anastomotic technique was associated with a low incidence of major airway complications; however, the absence of a comparison group precludes conclusions regarding its superiority over other continuous or interrupted reconstruction techniques [14,23]. The systematic application of a hybrid suturing technique, combining a continuous running suture for the membranous posterior wall with simple interrupted sutures for the cartilaginous ring using 4-0 polydioxanone monofilament, was intended to distribute tension evenly while limiting mucosal ischemia at the suture line [20]. In addition to suturing geometry, preserving anatomical structures such as the inferior pulmonary ligament during upper sleeve lobectomies has been described to reduce anastomotic tension and maintain anatomical stability of the remaining lung parenchyma [24]. Furthermore, routine coverage of the reconstructed bronchial and vascular suture lines with a vascularized autologous tissue flap, predominantly a pericardial fat pad or intercostal muscle flap, was intended to provide a physical barrier against broncho-vascular erosion and to promote healing of the reconstruction [25]; the individual contribution of the flap cannot be assessed in this study. These measures reflect our institutional reconstruction protocol; in this series, sleeve lobectomy was associated with an acceptable safety profile.

### 4.5. Nodal Status as the Dominant Prognostic Driver

Consistent with previous observations [26,27], pathological nodal involvement was the factor most strongly associated with overall survival in our exploratory multivariable model. Specifically, pathological N1 disease was associated with a more than two-fold higher hazard of death (HR = 2.46, *p* = 0.024), and pathological N2 disease with a doubled hazard (HR = 2.05, *p* = 0.050), while each additional involved lymph node was associated with a 15% higher hazard (HR = 1.15 per positive node, *p* = 0.017). Given the collinearity between these variables and the possibility of nodal understaging (Section 4.7), these estimates should be regarded as exploratory.

Furthermore, previous studies [28] support adjuvant chemotherapy in node-positive (N1/N2) disease to address systemic micrometastatic disease; data on adjuvant treatment were not available in our cohort.

### 4.6. Refined Surgical Decision Strategy and Nuanced Clinical Rationale

Our institutional approach to surgical selection balances anatomical radicality against physiological reserve using three groups of criteria (functional, lesional, and nodal; Section 3.7). This framework reflects our practice and is hypothesis-generating; it was not derived from or validated by the present data. Rather than a binary choice, both interventions have complementary roles.

Pneumonectomy provides maximal anatomical clearance for massive, centrally fixed tumors but is associated with a substantial physiological burden; in our unadjusted data, crude mortality was numerically higher after pneumonectomy (78.57% vs. 60.97%), which likely reflects, at least in part, more advanced disease. Sleeve lobectomy may be a reasonable option when functional or biological parameters—such as compromised cardiopulmonary reserve [7], advanced age, or cardiovascular comorbidities—make total lung resection hazardous. Patients presenting with central pulmonary malignancies frequently harbor substantial non-oncological complexities, chronic respiratory inflammation, and cardiovascular comorbidities that complicate clinical management and increase vulnerability to extensive parenchymal sacrifice [29]. In these settings, sleeve lobectomy is expected to avoid post-pneumonectomy respiratory impairment and acute right ventricular overload, although functional outcomes were not measured in this study.

In this cohort, when complete R0 resection was achieved, the surgical procedure was not significantly associated with overall survival after multivariable adjustment; given the sample size and the strong treatment-selection bias, this finding does not establish equivalent survival. Therefore, pneumonectomy is not the default choice for central tumors, but is used when parenchyma-sparing techniques cannot achieve complete R0 resection, such as in multi-lobar invasion or fixed main hilar involvement.

### 4.7. Study Limitations

This study is limited by its retrospective, single-center design and moderate sample size (*n* = 97). Additionally, baseline selection bias between surgical cohorts reflects real-world clinical practice where pneumonectomy is inherently required for more anatomically complex tumors. Multi-center prospective registries with standardized post-reconstruction surveillance are recommended to further validate long-term functional and quality-of-life outcomes. Furthermore, the mean number of harvested lymph nodes in our cohort (4.67 ± 1.90 in SL and 5.67 ± 2.67 in PN) represents individual lymph nodes rather than nodal stations. These yields fall below the optimal thresholds recommended by current IASLC guidelines for systematic nodal dissection [30]. This discrepancy reflects the real-world operational realities of a high-volume public institution during the studied period, where challenges in postoperative surgical specimen fragmentation and pathological sorting may occur, rather than an intentional omission of systemic staging [31]. This introduces a significant risk of nodal understaging (the “Will Rogers phenomenon”), which, combined with the lack of detailed data on postoperative adjuvant systemic therapy for pN1/pN2 patients [32,33], represents a major limitation in interpreting the long-term survival outcomes. Additionally, the multivariable Cox model was restricted to 89 complete cases, as 8 patients (8.2%) had missing tumor diameter or lymph node counts. With 67 events for 12 estimated parameters (approximately 5.6 events per parameter), the model is at risk of overfitting and its estimates are imprecise, so it should be regarded as exploratory; sensitivity analyses were not performed.

## 5. Conclusions

In conclusion, this retrospective, single-center observational study suggests that, in appropriately selected patients where an R0 parenchyma-sparing resection is technically feasible, sleeve lobectomy is associated with acceptable early morbidity and overall survival. Because of the strong treatment-selection bias inherent in real-world practice—where pneumonectomy is reserved for more anatomically extensive tumors—the two procedures cannot be considered directly comparable alternatives. In our multivariable analysis, after adjustment for baseline disease characteristics, the surgical procedure was not significantly associated with overall survival; this does not establish equivalence. Pathological nodal status and the number of involved nodes were the variables most strongly associated with overall survival in this exploratory model.

Both procedures have complementary roles. Pneumonectomy remains necessary for locally advanced anatomical configurations, fixed hilar invasion, or multi-lobar extension where R0 resection cannot be achieved otherwise. Sleeve lobectomy may be considered a parenchyma-sparing option whenever technically feasible and oncologically appropriate, particularly in patients with borderline functional reserve; this approach reflects our institutional practice and should be evaluated in larger, prospective studies.

## Figures and Tables

**Figure 1:**
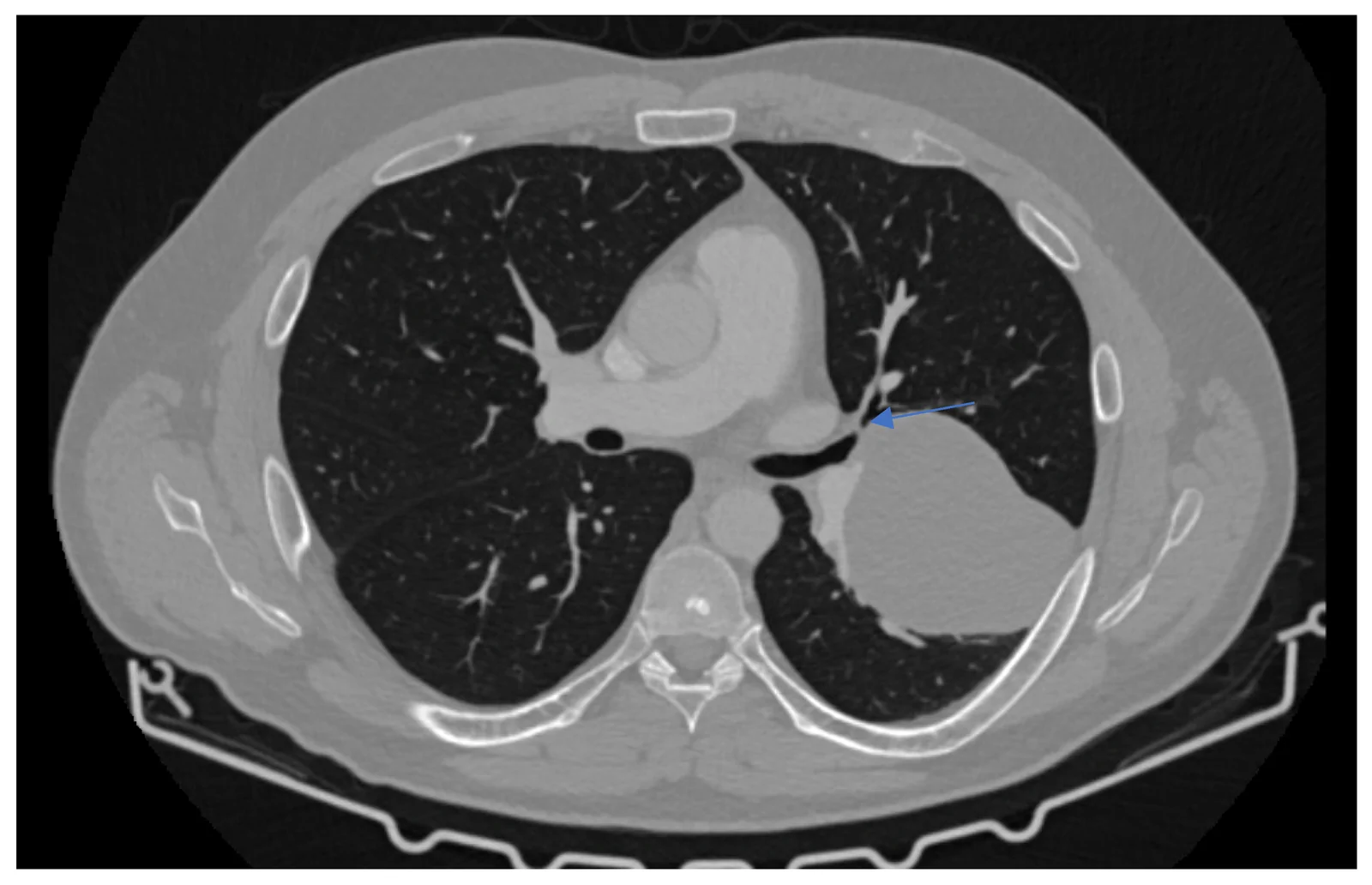
Chest computed tomography scan demonstrating a large left hilar mass with transfissural extension into both upper and lower lobes (blue arrow). The lesion maintains intimate contact with the descending thoracic aorta without evidence of direct invasion, alongside patent main pulmonary arteries and clear mediastinal lymph node stations.

**Figure 2:**
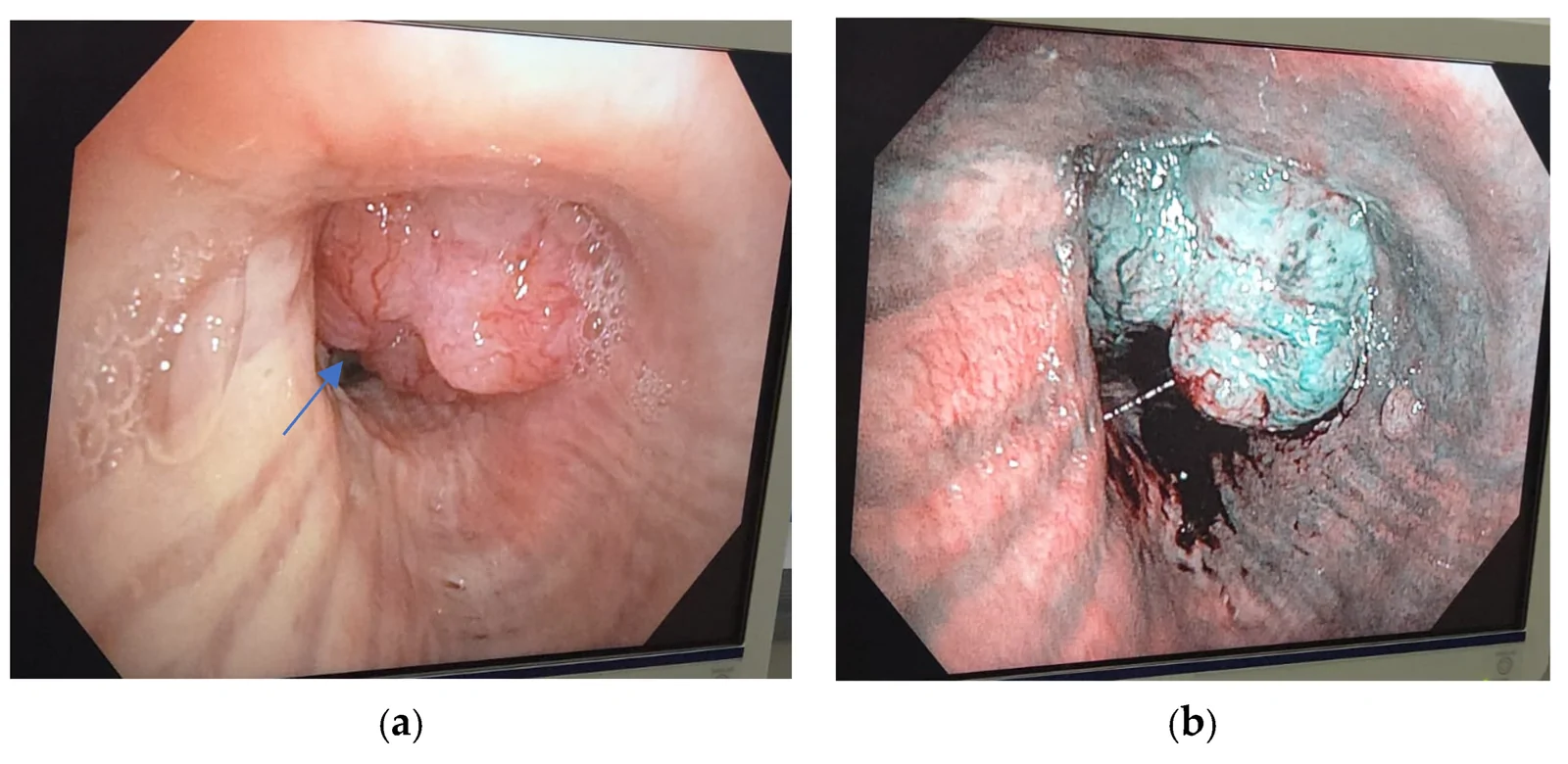
Preoperative flexible bronchoscopy evaluation of a central left upper lobe endobronchial tumor. (**a**) White-light bronchoscopy (WLB) showing a cauliflower-like, hypervascularized endobronchial lesion originating from the left upper lobe orifice, causing complete occlusion of the upper lobe and subtotal obstruction of the distal half of the left main bronchus starting at the level of the 4th cartilaginous ring (blue arrow). (**b**) Autofluorescence bronchoscopy (AFB)/fluorescence imaging highlighting altered mucosal fluorescence to help delineate proximal submucosal tumor margins (final margin status was determined by intraoperative frozen section).

**Figure 3:**
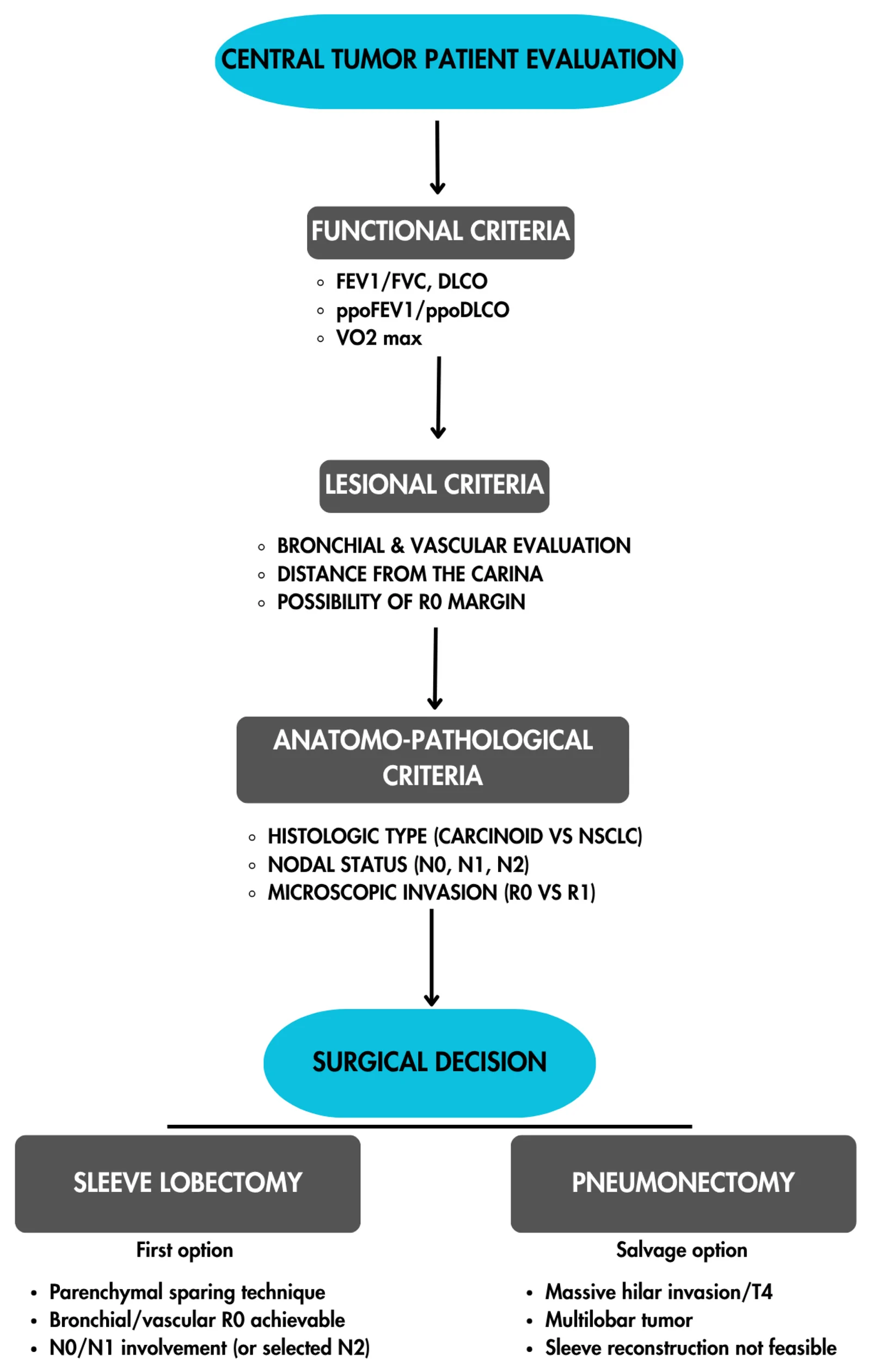
Conceptual framework reflecting institutional practice for surgical selection in centrally located non-small cell lung cancer (NSCLC). NB: In our practice, sleeve lobectomy is favored as the first-line surgical option based on functional, lesional, and nodal criteria, with pneumonectomy used when R0 sleeve reconstruction is not anatomically feasible. This framework reflects institutional practice and has not been validated.

**Table 1:** Patient Demographics and Clinical Characteristics.

Variables	Overall, *n* = 97	Sleeve Lobectomy Group (*n* = 41)	Pneumonectomy Group (*n* = 56)	*p*-Value
Age (years), Mean (SD)	58.00 (9.93)	58.95 (12.19)	57.30 (7.92)	0.45
Sex, n (%)				0.99
Female	26 (27%)	11 (27%)	15 (27%)	
Male	71 (73%)	30 (73%)	41 (73%)	
Histological Type, n (%)				0.78
Squamous cell carcinoma	45 (46%)	20 (49%)	25 (45%)	
Adenocarcinoma	37 (38%)	14 (34%)	23 (41%)	
Other type	15 (15%)	7 (17%)	8 (14%)	
T status, n (%)				**0.017**
T1	19 (20%)	13 (32%)	6 (11%)	
T2	40 (41%)	18 (44%)	22 (39%)	
T3	30 (31%)	9 (22%)	21 (38%)	
T4	8 (8.2%)	1 (2.4%)	7 (13%)	
N status, n (%)				0.55
0	33 (34%)	16 (39%)	17 (30%)	
1	25 (26%)	11 (27%)	14 (25%)	
2	39 (40%)	14 (34%)	25 (45%)	
Tumor Diameter (cm), Mean (SD)	5.01 (2.20)	4.67 (1.76)	5.25 (2.46)	0.20
Missing data (N/A)	6	3	3	
Harvested Lymph Nodes, Mean (SD)	5.26 (2.42)	4.67 (1.90)	5.67 (2.67)	**0.035**
Missing data (N/A)	3	2	1	
Involved Lymph Nodes, Mean (SD)	1.52 (2.18)	1.17 (1.51)	1.76 (2.54)	0.168
Missing data (N/A)	3	2	1	

Note: Statistical significance evaluated using Welch TwoSample t-test for continuous data, and Pearson’s Chi-squared test or Fisher’s exact test for categorical variables. Statistically significant values (*p* < 0.05) are highlighted in bold.

**Table 2:** Postoperative Complications According to the Clavien–Dindo Classification.

Clavien–Dindo Grade	Sleeve Lobectomy (*n* = 41), *n* (%)	Pneumonectomy (*n* = 56), *n* (%)	Clinical Presentation & Management
Grade I	4 (9.8%)	1 (1.8%)	Mild atelectasis/secretion retention managed with bedside chest physiotherapy and mucolytics.
Grade II	5 (12.2%)	14 (25.0%)	New-onset atrial fibrillation pharmacologically converted (*n* = 4 SL, *n* = 14 PN); partial vascular thrombosis managed with therapeutic anticoagulation (*n* = 1 SL).
Grade IIIa	4 (9.8%)	1 (1.8%)	Therapeutic flexible bronchoscopy for atelectasis/mucus plugs under local anesthesia (*n* = 2 SL, *n* = 1 PN); endoscopic balloon dilation for late bronchial stenosis (*n* = 2 SL).
Grade IIIb	1 (2.4%)	5 (8.9%)	Surgical re-intervention for anastomotic leak (*n* = 1 SL); surgical/stenting procedures under general anesthesia for mainstem bronchial stump fistula (*n* = 3 PN); open window thoracostomy/evacuation for chronic empyema (*n* = 2 PN).
Grade IVa	1 (2.4%)	3 (5.4%)	ICU admission requiring mechanical ventilation for early pulmonary edema (*n* = 1 SL); post-pneumonectomy pulmonary edema/early severe sepsis requiring mechanical ventilation and vasoactive support (*n* = 3 PN).
Grade IVb	0 (0.0%)	0 (0.0%)	Multiorgan dysfunction syndrome.
Grade V	1 (2.4%)	3 (5.4%)	30-day postoperative mortality.
Total patients with complications	16 (39.0%)	27 (48.2%)	Overall postoperative morbidity.

Note: Data are presented as mean +/− standard deviation, median (interquartile range), or count (percentage), as appropriate. ICU = Intensive Care Unit.

**Table 3:** Overall Survival Analysis.

Strata/Group	N Deaths (%)	Median Survival (95% CI) [Months]
Global Cohort	69/97 (71.13%)	36.0 (27.0 to 50.0)
Sleeve Lobectomy	25/41 (60.97%)	39.0 (33.0 to N/A)
Pneumonectomy	44/56 (78.57%)	28.5 (20.0 to 45.0)

Note: CI = Confidence Interval. Survival probabilities and median survival times were estimated using the Kaplan-Meier method.

**Table 4:** Multivariable Cox Proportional Hazards Regression Analysis for Overall Survival (*n* = 89).

Predictor Variable	Total (*N*)	Deaths (*n*)	Hazard Ratio (HR)	95% Confidence Interval (CI)	*p*-Value
Age (years)	89	67	1.00	0.97–1.03	0.831
Sex					
Female	23	16	Reference	—	—
Male	66	51	1.15	0.56–2.39	0.702
Surgical Procedure					
Sleeve Lobectomy (SL)	37	25	Reference	—	—
Pneumonectomy (PN)	52	42	1.31	0.74–2.31	0.349
Histological Subtype					
Adenocarcinoma	36	29	Reference	—	—
Squamous Cell Carcinoma	41	28	0.78	0.40–1.51	0.462
Other Types	12	10	1.60	0.70–3.66	0.270
Pathological T Stage					
T1	16	11	Reference	—	—
T2	37	26	0.59	0.25–1.37	0.218
T3	28	23	0.60	0.20–1.79	0.364
T4	8	7	1.50	0.50–4.49	0.469
Pathological N Stage					
N0	27	13	Reference	—	—
N1	24	19	2.46	1.12–5.40	**0.024**
N2	38	35	2.05	1.01–4.30	0.050
Tumor Diameter (cm)	89	67	1.12	0.96–1.30	0.155
Involved Lymph Nodes (count)	89	67	1.15	1.03–1.30	**0.017**

Note: HR = Hazard Ratio; CI = Confidence Interval; *N* = Number of patients. Multivariable Cox proportional hazards regression model incorporating all listed variables simultaneously (*N* = 89 evaluable patients; 8 patients excluded due to missing baseline covariates). Reference categories for categorical variables: Sex = Female; Surgical Procedure = Sleeve Lobectomy; Histological Subtype = Adenocarcinoma; Pathological T Stage = T1; Pathological N Stage = N0. Statistically significant *p*-values (*p* < 0.05) are highlighted in bold.

## Data Availability

The data presented in this study are available upon request from the corresponding author. The data are not publicly available due to institutional privacy and ethical restrictions regarding patient clinical records.

## Abbreviations

The following abbreviations are used in this manuscript:

NSCLC	Non-Small Cell Lung Cancer
SL	Sleeve Lobectomy
PN	Pneumonectomy
OS	Overall Survival
CI	Confidence Interval
HR	Hazard Ratio
R0	Complete Resection (microscopically negative margins)
R1	Incomplete Resection (microscopic residual disease)
CT	Computed Tomography
MRI	Magnetic Resonance Imaging
PET-CT	Positron Emission Tomography–Computed Tomography
PFT	Pulmonary Function Test
DLCO	Diffusing Capacity of the Lung for Carbon Monoxide
ppoFEV_1_	Predicted Postoperative Forced Expiratory Volume in 1 Second
ppoDLCO	Predicted Postoperative DLCO
CPET	Cardiopulmonary Exercise Testing
VO_2_max	Maximal Oxygen Consumption
EBUS-TBNA	Endobronchial Ultrasound-Guided Transbronchial Needle Aspiration
BPF	Bronchopleural Fistula
ARDS	Acute Respiratory Distress Syndrome
ESMO	European Society for Medical Oncology
AJCC/IASLC	American Joint Committee on Cancer/International Association for the Study of Lung Cancer
ICU	Intensive Care Unit
